# Initial Stroke Severity Is the Major Outcome Predictor for Patients Who Do Not Receive Intravenous Thrombolysis due to Mild or Rapidly Improving Symptoms

**DOI:** 10.5402/2011/947476

**Published:** 2011-07-27

**Authors:** Mu-Chien Sun, Tien-Bao Lai

**Affiliations:** ^1^Department of Neurology, Changhua Christian Hospital, Changhua 500, Taiwan; ^2^Department of Emergency Medicine, Changhua Christian Hospital, Changhua 500, Taiwan

## Abstract

Intravenous tissue plasminogen activator thrombolysis for stroke is still under use. A substantial proportion of excluded patients for mild or improving symptoms are dependent at discharge. We prospectively recruited 49 patients who did not receive thrombolysis because of mild or improving symptoms. 32 had favorable outcome (mRS ≤ 2) and 17 had unfavorable outcome (mRS > 2) at discharge. Comparisons were made between the two groups. Age was older (72.5 ± 10.0 versus 64.7 ± 13.2 years, *P* = 0.037), and initial National Institutes of Health Stroke Scale (NIHSS) score (5.7 ± 4.0 versus 2.2 ± 2.1, *P* < 0.001) was higher in the unfavorable group. Diastolic blood pressure was higher in the favorable group (98 ± 15 versus 86 ± 18  mmHg; *P* = 0.018). Atrial fibrillation (3.1 versus 23.5%; *P* = 0.043) and ipsilateral artery stenosis (21.9 versus 58.8%; *P* = 0.012) were more frequently found in the unfavorable group. Percentage of patients excluded from thrombolysis due to improving symptoms was higher in the unfavorable group (40.6 versus 82.4%; *P* = 0.005). Initial NIHSS score, but not other factors, was identified by logistic regression analysis as a major independent predictor for unfavorable outcome (OR 1.44; 95%CI, 1.03–2.02).

## 1. Introduction

Patients with minor or rapidly improving neurological signs are excluded from intravenous tissue plasminogen activator (tPA) thrombolysis for acute ischemic stroke in current American Heart Association/American Stroke Association guideline [1]. A substantial proportion of patients were excluded for these reasons [2–4]. Many among those patients were significantly dependent at discharge or unable to be discharged home [2, 5]. The aim of this study was to identify predictors for unfavorable outcome in this patient group.

## 2. Methods

Changhua Christian Hospital (CCH) is a medical center with 1.684 inpatient beds in western Taiwan, in an area with a population of 1.3 million. CCH is a participant of the nationwide Taiwan Stroke Registry (TSR) that enrolled acute stroke patients within 10 days after onset [6]. Code CCH-tPA was implemented for thrombolytic therapy in May 2008 in the hospital and activated 24 hours a day, seven days a week, at the triage by senior nursing staffs when both Cincinnati Prehospital Stroke Scale and a set of prescreen criteria are fulfilled. All patients who were excluded from intravenous thrombolysis due to mild or rapidly improving symptoms were prospectively recruited for analysis. Mild symptom was defined as National Institute of Health Stroke Scale (NIHSS) score lower than 4. Rapidly improving symptom was defined as regression of neurological symptoms between stroke onset and evaluation by the treating neurologist.

Any known history or newly found atrial fibrillation was recorded. Carotid duplex and/or magnetic resonant angiography (MRA) was used to identify vascular stenosis. Any ipsilateral extracranial or intracranial artery stenosis more than 50% was defined as artery stenosis.

Favorable outcome was defined as modified Rankin scale (mRS) score ≦ 2 at discharge. Unfavorable outcome was defined as mRS > 2 at discharge. Analyses for comparisons were made between groups with favorable and unfavorable outcomes. Independent predictors for unfavorable outcome were further identified by binary logistic regression analysis. Statistical analyses were performed with SPSS for Windows, version 13.0. The *χ* ^2^ and Fisher exact tests were used to compare dichotomous variables, and the unpaired, 2-tailed *t*-test was used to compare continuous variables.

## 3. Results

Between May 2008 and February 2010, 368 patients with acute ischemic stroke or TIA were admitted to our hospital within 3 hours of symptom onset. Fifty one were excluded from intravenous thrombolysis due to mild or rapidly improving symptoms. Two patients were excluded from this analysis: one left the hospital against medical advice, the other was severely disable before stroke due to femoral fracture. Forty-nine patients were included in this analysis. Among the included patients, 22 were excluded from thrombolysis due to mild symptoms and 27 due to improving symptoms. The initial NIHSS score was 1.9 ± 1.3 in those with mild symptoms and 4.6 ± 3.9 in those with improving symptoms. The difference of initial NIHSS score between the two groups was not statistically significant (*P* = 0.237).

Among 49 patients, 32 had favorable outcome, and 17 had unfavorable outcome at discharge (Table 1). Age was older (72.5 ± 10.0 versus 64.7 ± 13.2 years, *P* = 0.037), and initial NIHSS score (5.7 ± 4.0 versus 2.2 ± 2.1,* P* < 0.001) was higher in the unfavorable group. Diastolic blood pressure was significantly higher in the favorable group (98 ± 15 versus 86 ± 18 mmHg; *P* = 0.018). Electrocardiography and vascular study were performed in high proportion of patients in both groups. Atrial fibrillation (3.1 versus 23.5%; *P* = 0.043) and ipsilateral artery stenosis (21.9 versus 58.8%; *P* = 0.012) were more frequently found in the unfavorable group. Percentage of patients excluded from thrombolysis due to improving symptoms was higher in the unfavorable group (40.6 versus 82.4%; *P* = 0.005). 

Further logistic regression analysis identified initial NIHSS score, but not other factors, as an independent predictor for unfavorable outcome (OR 1.44; 95%CI, 1.03–2.02) (Table 2, model 1). Using dichotomous NIHSS score (at 3) in the regression analysis demonstrated high risk of unfavorable outcome (OR 5.95; 95%CI, 1.10–32.12) for patients with initial NIHSS score  ≧  3. No other factors independently predicted unfavorable outcome, including improving symptom (Table 2, model 2).

## 4. Discussion

In tPA thrombolytic therapy for acute ischemic stroke, exclusion criteria “mild neurological impairment” and “rapidly improving symptoms” were not clearly defined and left to the clinicians' interpretation. A proportion of stroke patients are not receiving thrombolytic therapy due to these exclusion criteria. Some studies aimed at identifying patients who are excluded but will benefit from the treatment [7, 8]. A post hoc subgroup analyses from the NINDS rt-PA Stroke Study group showed that no difference was detected in the beneficial effects of tPA in patients with 5 different definitions of minor stroke syndromes compared to the overall treatment effects in the entire cohort [7]. 32 patients with mild symptoms with aphasia representing the most common symptom were treated with intravenous thrombolysis. Outcome was favorable. Patients with mild but disabling symptoms were suggested to be treated with tPA regardless of their baseline NIHSS score [8]. 

An operational definition of “mild neurological impairment” seems not to be feasible based on NIHSS score. However, an international survey on the inclusion and exclusion criteria for tPA thrombolysis reached a consensus on a minimal NIHSS score of 2 to 3 to warrant treatment [9]. Our study demonstrated that patients with initial NIHSS score above this limit had 5.95 times risk to have unfavorable outcome. On the contrary, consensus was not reached in the survey on rapidly improving symptoms. A study on patients who were excluded from tPA treatment due to mild or improving symptoms showed that patients who had 4-point improvement in NIHSS score and were excluded from tPA treatment were more likely to have subsequent neurological worsening. However, no single variable at presentation was identified to be associated with death or lack of home discharge [5]. In our study, we identified initial NIHSS score as the sole predictor for unfavorable outcome in patients who were excluded from tPA treatment because of mild or rapidly improving symptoms. 

A study on thrombolytic treatment to 19 acute stroke patients with rapid early improvement of neurological deficit showed no neurological deterioration during hospitalization and good outcomes. The NIHSS score before treatment was 5 (4 to 6) [10]. A high proportion of excluded patients in our study (34.7%) had unfavorable outcome. Treatment for patients with higher initial NIHSS score, regardless of regressing symptoms, seems to be justified. Thus, we propose that “rapidly improving symptoms” may be removed from the exclusion criteria for intravenous tPA thrombolysis in stroke patients. Further studies are required to identify a feasible definition of “mild symptoms” and justify a cut-off point of initial NIHSS score for thrombolytic therapy.

## Figures and Tables

**Table 1:** Characteristics of patients in the groups of favorable and unfavorable outcome.

	Favorable	Unfavorable	*P*
No	32	17	
Age, years	64.7 ± 13.2	72.5 ± 10.0	0.037
Female sex	10 (31.3)	9 (52.9)	0.138
Initial presentations			
NIHSS	2.2 ± 2.1	5.7 ± 4.0	< 0.001
Improving symptoms*	13 (40.6)	14 (82.4)	0.005
Systolic BP, mmHg	170 ± 26	162 ± 26	0.261
Diastolic BP, mmHg	98 ± 15	86 ± 18	0.018
Glucose, mg/dL	158 ± 64	151 ± 50	0.724
Risk factors			
Atrial fibrillation	1 (3.1)	4 (23.5)	0.043
Artery stenosis	7 (21.9)	10 (58.8)	0.012
Hypertension	23 (71.9)	14 (82.4)	0.417
Diabetes	9 (28.1)	8 (47.1)	0.185
Previous stroke	5 (15.6)	4 (23.5)	0.700
Smoking habit	14 (43.8)	5 (29.4)	0.327
Outcome at discharge			
NIHSS	1.4 ± 1.3	5.3 ± 3.3	< 0.001
mRS	1.2 ± 0.6	3.4 ± 0.5	< 0.001
Barthel index	95.7 ± 8.4	57.7 ± 23.9	< 0.001
Examinations			
MRA	16 (50.0)	12 (70.6)	0.166
Carotid duplex	28 (87.5)	17 (100.0)	0.248
MRA or duplex	29 (90.6)	17 (100.0)	0.542
EKG	32 (100.0)	17 (100.0)	—

*Number of patients excluded from thrombolysis due to improving symptoms. Continuous variables are median ± standard deviation.

Numbers in parenthesis denote percentage.

**Table 2:** Logistic regression analyses for unfavorable outcome.

	Odds ratio	95% C.I.	*P*
*Model 1*			
NIHSS score	1.44	1.03–2.02	0.034
Age	1.03	0.95–1.12	0.493
DBP	0.96	0.91–1.02	0.229
Atrial fibrillation	11.56	0.63–211.75	0.099
Artery stenosis	1.69	0.29–9.79	0.561
Improving symptom	2.29	0.37–14.02	0.372

*Model 2*			
NIHSS ≧ 3 versus < 3	5.95	1.10–32.12	0.038
Age	1.04	0.95–1.12	0.459
DBP	0.96	0.91–1.02	0.216
Atrial fibrillation	5.21	0.24–110.98	0.290
Artery stenosis	2.57	0.45–14.61	0.285
Improving symptom	2.94	0.49–17.59	0.237
